# Evaluation of fluorescence-based viability stains in cells dissociated from scleractinian coral *Pocillopora damicornis*

**DOI:** 10.1038/s41598-022-19586-7

**Published:** 2022-09-12

**Authors:** Liza M. Roger, Yaa Adarkwa Darko, Tytus Bernas, Frances White, Monsurat Olaosebikan, Lenore Cowen, Judith Klein-Seetharaman, Nastassja A. Lewinski

**Affiliations:** 1 Chemical and Life Science Engineering, Virginia Commonwealth University, Richmond, VA USA; 2 School of Molecular Sciences, Arizona State University, Phoenix, AZ USA; 3 Anatomy and Neurobiology, Virginia Commonwealth University, Richmond, VA USA; 4 Department of Computer Sciences, Tufts University, Boston, MA USA; 5 College of Health Solutions, Arizona State University, Phoenix, USA

**Keywords:** Cell biology, Environmental sciences, Ocean sciences, Nanoscience and technology

## Abstract

The application of established cell viability assays such as the commonly used trypan blue staining method to coral cells is not straightforward due to different culture parameters and different cellular features specific to mammalian cells compared to marine invertebrates. Using *Pocillopora damicornis* as a model, we characterized the autofluorescence and tested different fluorescent dye pair combinations to identify alternative viability indicators. The cytotoxicity of different representative molecules, namely small organic molecules, proteins and nanoparticles (NP), was measured after 24 h of exposure using the fluorescent dye pair Hoechst 33342 and SYTOX orange. Our results show that this dye pair can be distinctly measured in the presence of fluorescent proteins plus chlorophyll. *P. damicornis* cells exposed for 24 h to Triton-X100, insulin or titanium dioxide (TiO_2_) NPs, respectively, at concentrations ranging from 0.5 to 100 µg/mL, revealed a LC50 of 0.46 µg/mL for Triton-X100, 6.21 µg/mL for TiO_2_ NPs and 33.9 µg/mL for insulin. This work presents the approach used to customize dye pairs for membrane integrity-based cell viability assays considering the species- and genotype-specific autofluorescence of scleractinian corals, namely: endogenous fluorescence characterization followed by the selection of dyes that do not overlap with endogenous signals.

## Introduction

Cytotoxicity, the potential to cause cell death, is measured via different endpoints^1^. These endpoints can include membrane integrity, mitochondrial function, proliferation, and apoptosis *versus* necrosis. It is important to systematically combine multiple endpoints, such as membrane integrity and cell death mechanism, to gain insight into the potential pathways involved in cellular toxicity. The most common way to measure these endpoints is by using colorimetric or fluorometric assays which involve the addition of an indicator dye. Many indicator dyes have been developed for different endpoints and finding the right type of dye hinges on the cell model system studied, desired throughput, instruments available for analysis, and cost of reagents^2^. Fluorescent indicator dyes (fluorophores) are most commonly used in biochemical and cell-based assays in vertebrate cell culture since fluorescence is more sensitive^3^ and fluorescence is utilized in many instruments (microscopy, spectroscopy, flow cytometry). Fluorophores used to assess cytotoxicity mostly emit visible light in the blue, green and red color bands. Table 1 includes common fluorophores used to assess membrane integrity and distinguish between live and dead cells when conducting viability assays.

**Table 1:** Fluorophores tested on coral cells and conclusions.

Fluorophore λ_ex_/λ_em_ (nm)	FC*	Type	Findings
Hoechst 33258, 352/461 (blue)	40 µM	Membrane permeable, non-toxic	Does not penetrate the cells well
Hoechst 33342, 361/497 (blue)	40 µM	Membrane permeable, non-toxic	Bright stain but needs a minimum of 30 min incubation at 25 °C
NucBlue, 360/460 (blue)	NA**	Membrane permeable, non-toxic	Bright stain, Hoechst 33342 is active agent here
Calcein AM blue, 408/450 (blue)	2 µM	Membrane permeable, non-toxic	Stains but weakly fluorescent
Calcein AM, 496/515 (green)	2 µM	Membrane permeant	Overlaps with GFP, cannot be deconvolved
Ethidium homodimer, 528/617 (red)	4 µM	Membrane impermeant	Efficient and visible staining but must be manually deconvolved from chlorophyll spectrum
POPO-3 iodide, 534/570 (orange)	2 µM	Membrane impermeant, non-toxic	Cannot be deconvolved using the RFP filter cube but laser scanning microscopy will work
SYTOX Red, 540/658 (red)	0.2 µM	Membrane impermeant, non-toxic	Efficient and visible staining but must be manually deconvolved from chlorophyll spectrum
SYTOX Orange, 547/570 (orange)	0.2 µM	Membrane impermeant, non-toxic	Efficient and visible staining but can stain contamination if present
BOBO-3 iodide, 570/602 (orange)	2 µM	Membrane impermeant, non-toxic	Cannot be deconvolved using the RFP filter cube but laser scanning microscopy will work
Propidium iodide, 585/617 (red)	1 µg/mL	Membrane impermeant, toxic ≥ 24 h	Efficient and visible staining but must be manually deconvolved from chlorophyll spectrum

*FC final concentration in cell suspension.

**Not specified by vendor.

The need for cellular-scale understanding of mechanisms such as symbiosis, calcification, wound healing and bleaching is increasing as reef-building corals (Scleractinia) are more than ever under stress from rising sea surface temperatures, ocean acidification, diseases, pollution and habitat loss^4^. While many studies have focused on colony-scale or polyp-scale dynamics, cellular studies are less common because the scientific community has only reported generating axenic, immortal scleractinian coral cell cultures twice since the early 1990s (see^5,6^) and concerns remain regarding contamination and cell identification as our knowledge of the coral holobiont grows^4,7^. The further development of scleractinian coral cell cultures requires quantitative assessment of survivorship through basic live and dead cell counts. Survivorship being critical to determining culture method success, it is important to have a clear approach for quantifying population viability. The use of trypan blue, a colorimetric, cell impermeant stain, to quantify cell death is the most reported method in relation to coral cell cultures despite its limitations. The main issue with trypan blue is its capacity to bind to proteins in the cell suspension, not just that released by dead cells. This results in blue aggregates that hinder the cell count. There is evidence that trypan blue can be toxic in the case of longer incubation times^8^. Other colorimetric dyes that have been tested on coral cells include cell impermeant stains such as Evans blue and amido black, as well as vital stains such as neutral red and toluidine blue^9,10^. Nevertheless, the range of imaging and measurement techniques for colorimetric stains are limited in comparison to fluorescent stains^9^. Work on symbiotic algae showed that the nucleic acid stain SYTOX Green (λ_ex_ 504 nm, λ_em_ 523 nm) and propidium iodide successfully labelled nuclei of dead Symbiodinium clade A and B cells^9^ and *Symbiodinium* sp. cells isolated from *Stylophora pistillata*^11,12^. However, excitation and emission wavelengths of STYOX Green overlap with green fluorescent protein which is present in many coral host species and certain species of symbiotic algae (e.g. *Breviolum minutum*, formerly Clade B).

**Figure 1:**
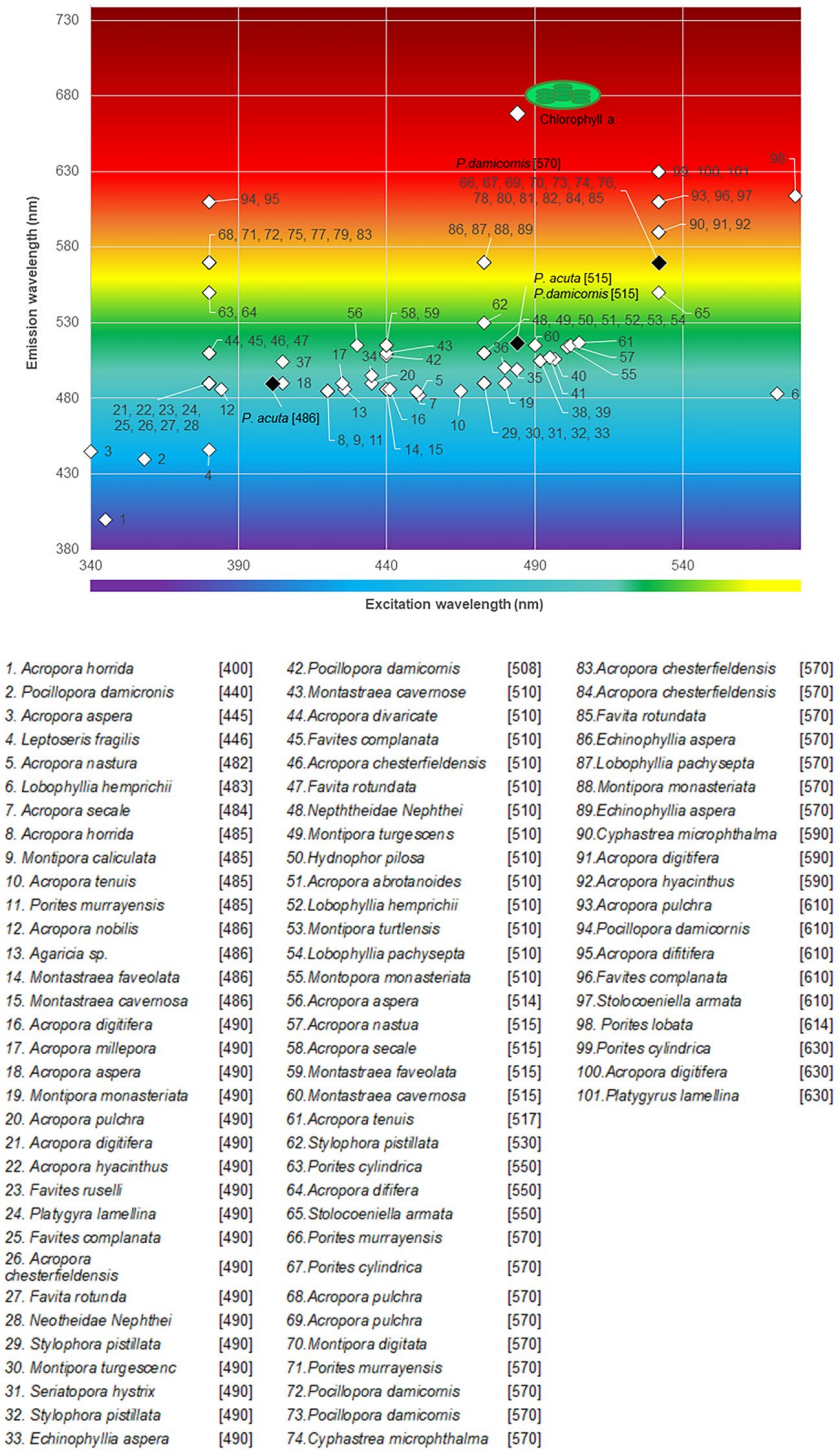
101 Coral chromophores. Selection of 101 coral chromophores excitation (λ_ex_) and emission (λ_em_) wavelengths reported in 10 different coral families (43 scleractinian coral species) and measured in *Pocillopora damicornis* (this study). Coral species are listed with number key corresponding to the graph and [emission wavelength] detected (and reported in literature). References are as follow: (1–3, 8–9, 11, 17, 20, 35–36, 38)^49^; (4)^50^; (5, 7, 10, 19, 55–57, 61)^51^; (6)^52^; (12)^53^; (13)^19^; (14–15, 58–59)^54^; (16, 60)^55^; (18, 37)^56^; (21–33, 44–54, 62–97, 99–101)^57^; (39)^58^; (34, 41)^17^; (40)^18^; (42)^59^; (43)^60^, (98)^20^.

Fluorescent proteins extracted from marine invertebrates, especially Cnidarians, have helped advance biological research thanks to their capacity to serve as molecular markers and biosensors^13^. The green fluorescent protein (GFP) was first characterized in the jellyfish *Aurelia victoria* (*Aequorea victoria*)^14^ and has now become one of the most useful tools in modern science and medicine^15^*.* Multiple coral chromophores have been identified so far including cyan fluorescent protein (λ_ex_ 404–477 nm, λ_em_ 485–495 nm), green fluorescent protein (λ_ex_ 478–508 nm, λ_em_ 500–520 nm) and red fluorescent protein or symbiotic chlorophyll (λ_ex_ 560–589 nm, λ_em_ 576–595 nm), and non-fluorescent pink-purple-blue (λ_ex_ 560–588 nm)^16–18^ (Fig. 1). Yellow (λ_ex_ 425–550 nm, λ_em_ 525–570 nm) and bright far-red (λ_ex_ 578 nm, λ_em_ 617 nm) fluorescent proteins are also present in corals but appear to be specific to *Agaricia* sp.^19^ and *Porites lobata* (pink phenotype)^20^. It should be noted that, beyond chlorophyll, other endogenous fluorescent signals from the coral microbiome (symbiotic algae included) were not considered here due to the difference in scale at which they might interfere relative to that at which the work was performed. The use of fluorophores in cells with endogenous fluorescent proteins, such as scleractinian coral cells, presents a challenge because fluorophore emission wavelengths can overlap with that of the endogenous fluorescence emitted by fluorescent proteins or symbiotic algae cells (chlorophyll) hosted within coral cells, thereby confounding the signal.

**Figure 2:**
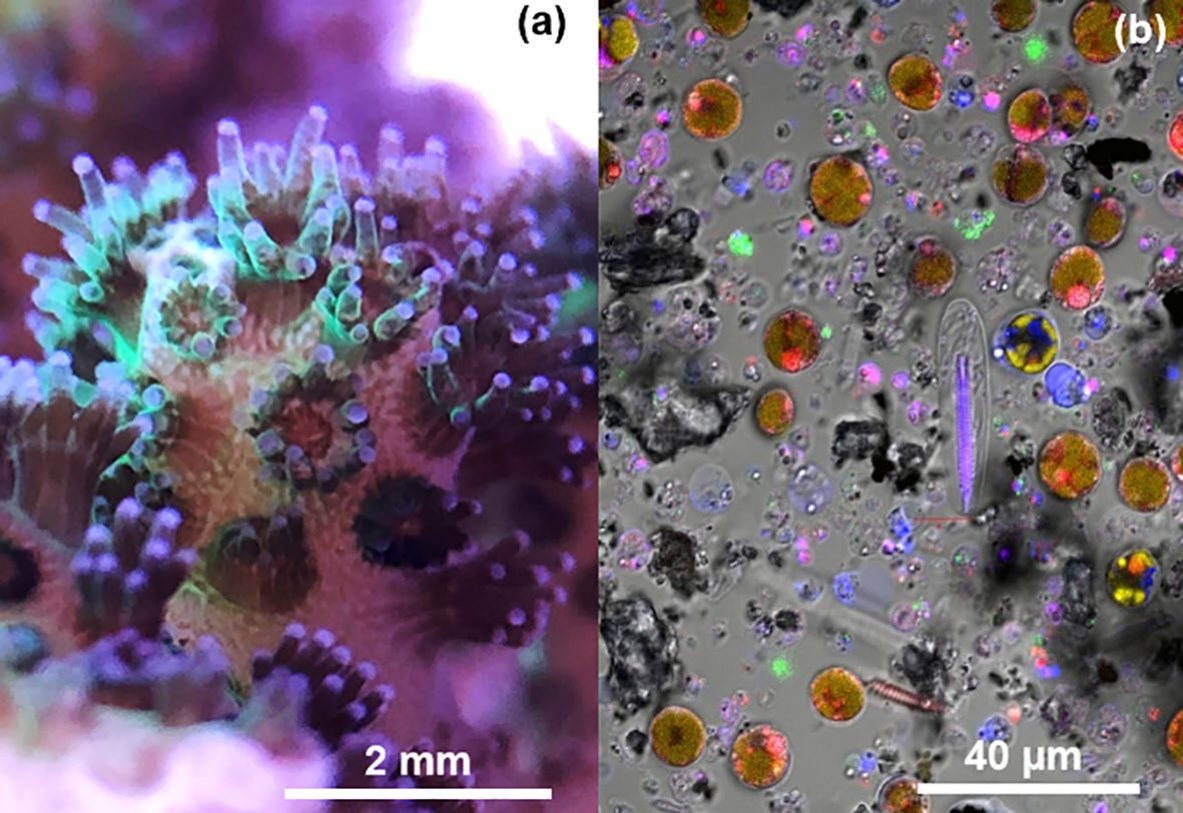
Photograph of *Pocillopora damicornis* in aquarium with polyps displaying the green fluorescent phenotype (**a**) and cells dissociated from *P. damicornis* stained with ReadyProbes cell viability imaging kit (red: propidium iodide for dead cells, blue: NucBlue for all cells) imaged under confocal laser scanning microscope (Zeiss LSM 710, VCU Microscopy Core) [other colors are as follows: yellow for chlorophyll fluorescence and green for endogenous GFP, DIC set for contrast on center cell] (**b**). The most recognizable cell types are the symbiotic dinoflagellates (golden-brown rounded cells, color generated from overlay of yellow and red) and the nematocytes (oblong cells with bottle brush-like internal structure). Other cell types cannot be identified from morphological characteristics.

The objective of this study was to tailor a fluorescence-based dye exclusion assay for cell viability and cytotoxicity assessments to scleractinian coral research by taking into consideration the endogenous fluorescent signal (i.e., fluorescent stains to differentiate dead cells from live cells that are detectable in the presence of endogenous cellular fluorescence). To this end we tested established membrane integrity assays developed for vertebrate cells on cells dissociated from *Pocillopora damicornis* (Fig. 2, mixed cells: coral and symbiotic algae) and investigated the overlap between assay fluorophores and endogenous proteins. We then used the resulting protocol to verify the cell viability of dissociated coral cells and test the cytotoxicity of three representative classes of molecules, namely the small molecule Triton X-100, the protein insulin, and a representative engineered nanoparticle, titanium dioxide, as a proof-of-concept and first assessment of the effects of different types of molecules on coral cell physiology.

## Results

**Figure 3:**
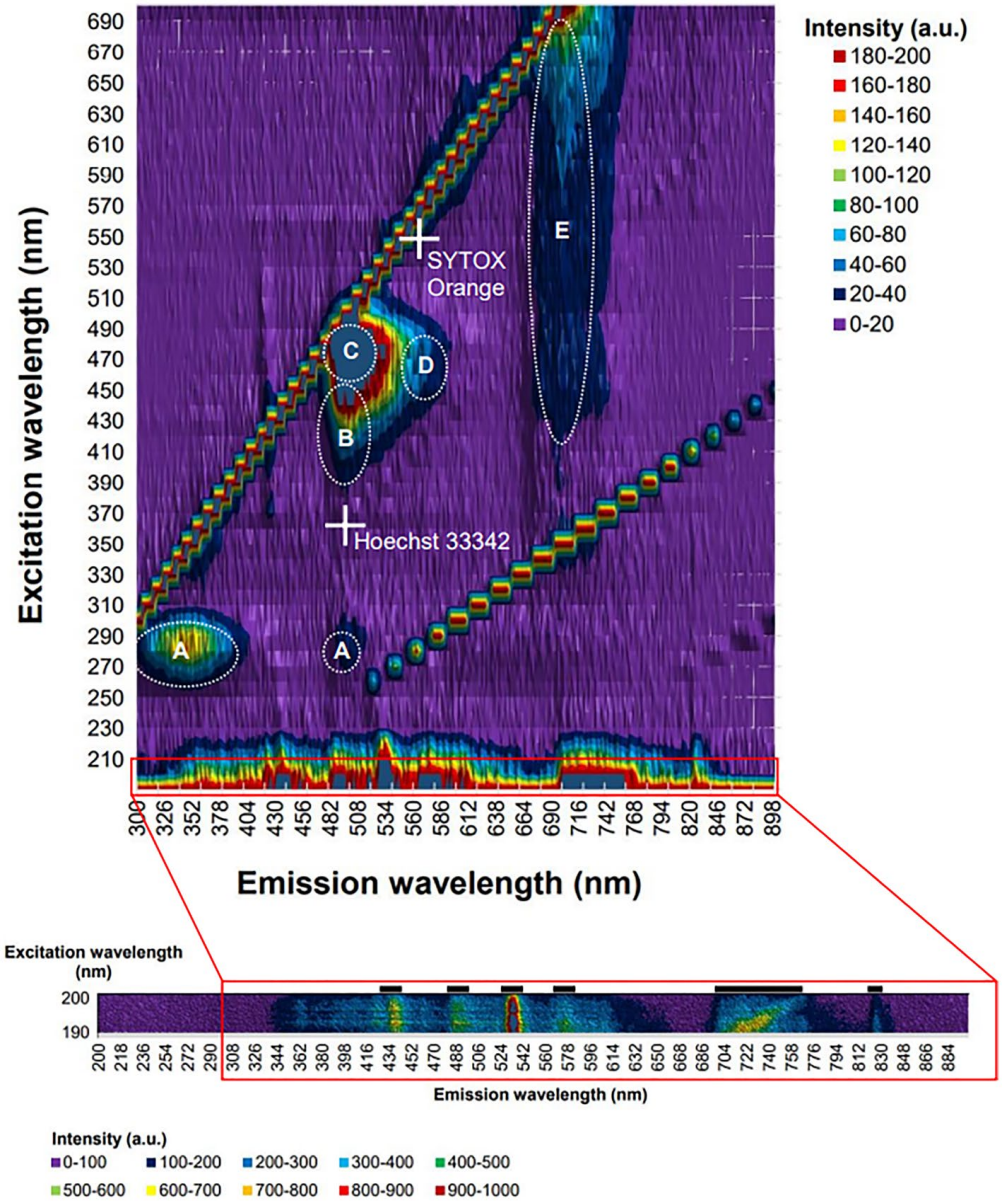
Excitation–emission matrix for *Pocillopora damicornis* from UV/VIS-fluorescence spectrophotometry. Five emission peaks are present: tryptophan (**A**), cyan fluorescent protein (**B**), green fluorescent protein (**C**), red fluorescent protein (**D**) and chlorophyll (**E**). Insert shows high resolution fluorescence matrix under deep UV excitation (190 to 200 nm), which also contains the same five emission peaks. Note the chlorophyll fluorescence presents a wide emission band from ~ 700 nm, which is well documented^61^. White crosses represent excitation and emission wavelengths of the dye pair used for membrane integrity assessment (SYTOX Orange and Hoechst 33342).

Tissue of scleractinian coral *Pocillopora damicornis* (green phenotype, Fig. 2) was dissociated from the skeleton using calcium and magnesium-free seawater incubation for 1 h as described in our earlier publication^4^. The mixture of cells was washed and resuspended in complete culture medium (CM, see “Coral cell dissociation and culture” section for details). The resulting cell suspensions were then observed under a confocal laser scanning microscope and analyzed using a fluorimeter to measure the autofluorescence of *P. damicornis*. The excitation–emission matrix (EEM, Fig. 3) shows five emission peaks representing tryptophan (λ_ex_ 280 nm, λ_em_ 350 nm, A), cyan fluorescent protein (B), green fluorescent protein (C), red fluorescent protein (D) and the endosymbionts’ chlorophyll (E). The lambda scans confirmed these fluorescent signals (Supplementary Materials S1).

**Figure 4:**
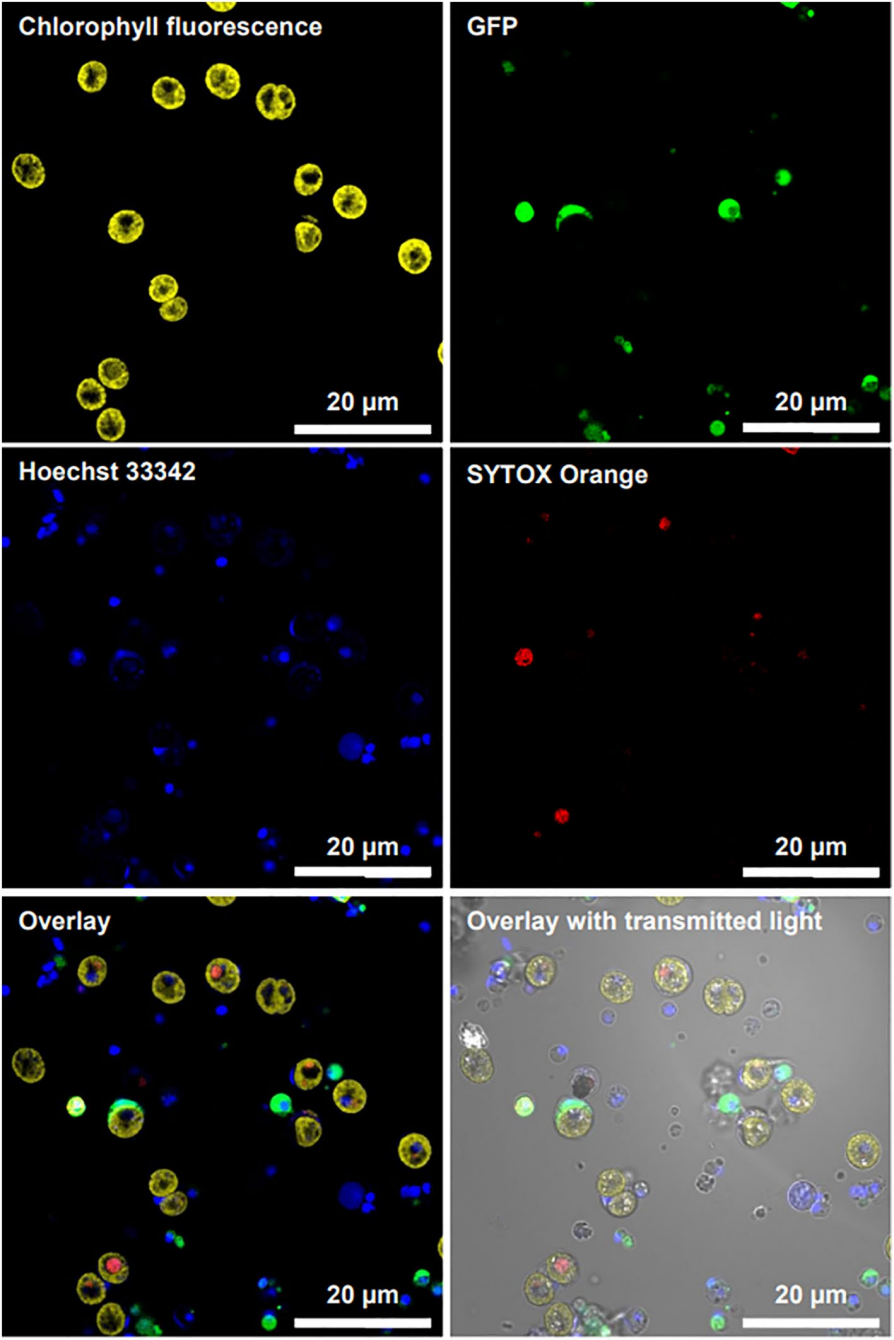
Live cell confocal fluorescence laser scanning microscopy images of *Pocillopora damicornis* cells (Zeiss LSM 710, VCU Nanomaterial Characterization Core). Cells were co-stained with SYTOX Orange (0.2 µM) and Hoechst 33342 (40 µM) for 30 min. Magnification = 63×.

The fluorophores tested on the *P. damicornis* cells were Hoechst 33258 (blue, all cells), Hoechst 33342 (blue, all cells), NucBlue (blue, all cells), Calcein blue AM (blue, live), Calcein AM (green, live), Ethidium homodimer (red, dead), POPO-3 iodide (orange, dead), SYTOX Red (red, dead), SYTOX Orange (orange, dead), BOBO-3 iodide (orange-red, dead), and Propidium iodide (red, dead). The choice of fluorophores to test was guided by emission spectra, staining mechanism and detection capabilities available (confocal laser scanning microscope and microplate reader equipped with the following filter cubes: DAPI, GFP, RFP and Texas Red). Furthermore, a pair of dyes with one indicating dead cells and the other indicating live or all cells was sought for increased accuracy. Calicoblastic cells, i.e. calcifying cells, are ~ 10× smaller than symbiotic gastrodermal cells and nematocytes (also referred to as cnidocytes, stinging cells) Fig. 2. Without a dye pair combination for viability, cell density can easily be underestimated and smaller cells be inadvertently disregarded as debris. Of these fluorophores, the best combination was Hoechst 33342 and SYTOX Orange with the Hoechst 33342 staining all cells and the SYTOX Orange only the dead cells, leaving the red channel for endosymbiont detection and the green channel for endogenous GFP (Fig. 4, see “Live/dead assay” section for details). The outcomes of staining for all fluorophores tested are summarized in Table 1.

As described in Table 1, several established live/dead stains are ill-suited to coral cells because of spectral overlap with endogenous signals. When using bandpass filters, the emission from the cell impermeant stains propidium iodide and ethidium homodimer cannot be separated from endosymbiont chlorophyll fluorescence to mark dead cells. The nucleic acid stain SYTOX Orange (λ_ex_ 547 nm, λ_em_ 570 nm), also a cell impermeant stain, penetrates cells with compromised plasma membrane and has an emission wavelength further away from that of chlorophyll. The SYTOX line of dead stains is available in different colors, which could be useful in coral samples with emission spectra near 570 nm, as reported for number of Acroporidae, Poritidae, and Faviidae corals (Fig. 1).

### Cytotoxicity assay

In order to test the cytotoxicity of titanium dioxide nanoparticles (TiO_2_ NPs) and insulin, cell mixtures collected from *P. damicornis* were plated on 96 well-plates coated with poly-d-lysine at a density of ~ 400,000 cells per well with complete medium. After a 1 h rest period, the cells were exposed to concentrations of insulin and TiO_2_ NPs ranging from 0.5 to 100 µg/mL in culture medium. Triton X-100 was used as positive control. Cell survival was measured after 4 h of exposure using the MTS and LDH assays and after 24 h of exposure using the Hoechst 33342—SYTOX Orange dye pair.

Two colorimetric assays were tested here: the MTS and LDH assays. The MTS (3-(4,5-dimethylthiazol-2-yl)-5-(3-carboxymethoxyphenyl)-2-(4-sulfophenyl)-2H-tetrazolium) and LDH (lactate dehydrogenase) assays are commonly performed in combination. Although reliable and simple, limitations of the MTS and LDH assays are that serum and other compounds in the culture media can introduce distortions or background^21,22^.

The MTS assay did not yield satisfactory results after 1 h at 25 °C, the incubation time recommended for mammalian cells. The expected reaction is a color change from the yellow MTS tetrazolium as it is converted by cell metabolism to the purple/brown formazan. No color change was also measured with longer incubation times of 6, 12, 24 and 48 h at 25 °C. The absorbance measurements of the failed MTS assays are not presented. The LDH assay successfully measured the presence of lactate dehydrogenase in the media taken from the exposed *P. damicornis* cell. However, the results were variable and the media-only control was not consistent throughout the experiments (Supplementary Materials S.2).

**Figure 5:**
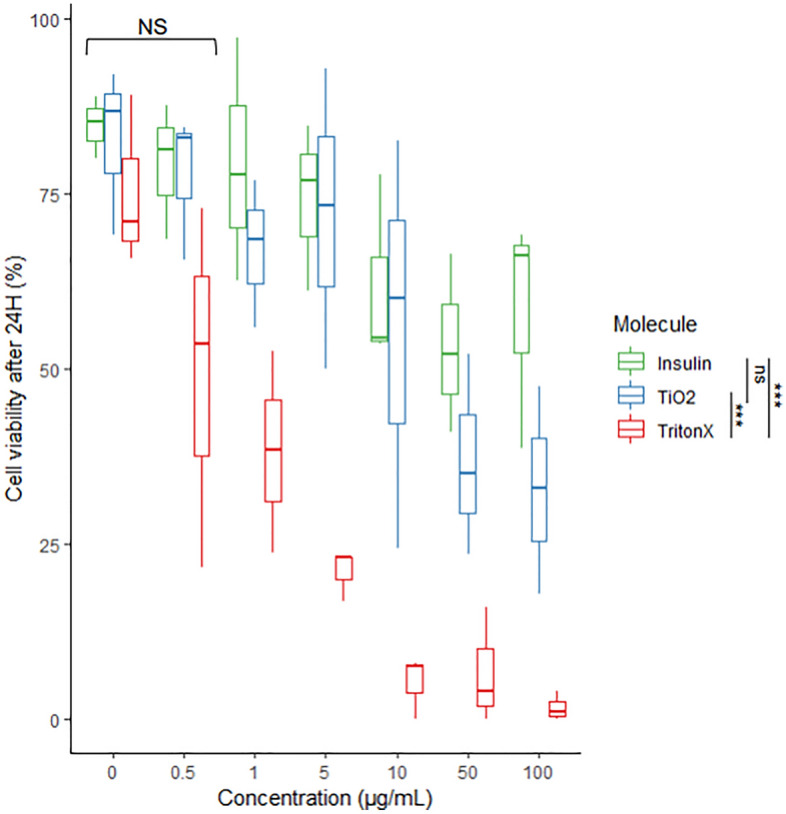
Coral cell viability after 24 h exposure to concentrations of Triton X-100 (positive control, cell lysate), titanium dioxide (TiO_2_) and insulin between 0.5 and 100 µg/mL. The boxes summarize the data with median (bold line) and outliers (whiskers). The different molecules tested yielded statistically significant results between 1 and 100 µg/mL but not between 0 and 0.5 µg/mL (NS). The control used (Triton X-100) yielded significantly different results compared to TiO_2_ and insulin, confirming its role as positive control. Further statistical analyses can be found in Supplementary Materials S.2.

The measurements based on the Hoechst 33342—SYTOX Orange dye pair showed TiO_2_ NPs reduces *P. damicornis* cell viability at concentrations above 10 µg/mL with a calculated lethal concentration 50% (LC50) of 6.21 µg/mL (95% confidence interval, CI from 2.39 to 15.6) after 24 h of exposure. Insulin also reduced *P. damicornis* cell viability (LC50 33.9 µg/mL, 95% confidence interval, CI from 9.16 to 1736) for concentrations between 10 and 100 µg/mL after 24 h of exposure (Fig. 5). Statistical analysis (two-way ANOVA with blocking and Tukey post-hoc, see “Statistical analysis” section) revealed significant differences between the control (0 µg/mL) and concentrations above 1 µg/mL for all treatment (Supplementary Materials S.2). Significant differences were also found between the positive control treatment (Triton X-100) and TiO_2_, and insulin, confirming its efficacy as positive control. No significant difference was found between the TiO_2_ and insulin treatments (Fig. 5, Supplementary Materials S.2).

## Discussion

Mammalian cell-based assays are poorly adapted to in vitro work with scleractinian coral cells. This is in part due to the diversity and abundance of endogenous fluorescence present in reef-building corals but also due to factors such as the lack of cell attachment, the salinity (~ 35‰) or strong ionic (~ 0.7 M) nature of seawater, and other unknowns. Indeed, as our knowledge of coral cell physiology and function grows, so will the diversity of coral-specific assays. Coral cell viability assessment is key to developing other assays as cell viability is one of the most straightforward endpoints of in vitro research. In this study we developed a framework for coral scientists to tailor fluorescence-based membrane integrity assays to the coral species phenotype of their choice. This method first involves accurately determining the different fluorescent signals emitted by the coral species genotype and finding fluorescent dyes that do not overlap or that can easily be deconvolved. The membrane integrity-based dye pair Hoechst 33342-SYTOX Orange, avoids the endogenous fluorescent signals of *P. damicornis* cells, allows for determination of cell viability independent of a reference (control) sample, and allowed us to test the toxicity of TiO_2_ and insulin in vitro applied to cells dissociated for scleractinian coral *P. damicornis* (green phenotype) for the first time.

Two viability assays common in mammalian research, the MTS and LDH assays, were also tested yielding unsatisfactory results. Cell viability has previously been tested using the MTT tetrazolium reduction assay^23^. Both MTT and MTS are tetrazolium-based dyes; however, MTS relies on an intermediate electron acceptor for reduction to formazan. In addition, the background absorbance from the culture media and MTS alone at the measurement wavelength of 490 nm is higher than the background absorbance for the MTT assay^24^. The inconsistencies in the LDH assay results on the other hand, might be due to the presence of other dehydrogenases, such as opine dehydrogenases which are functionally analogous to lactate dehydrogenase, being used to regenerate NAD^+^ in many invertebrates^25^. *Montipora capitata*, another scleractinian coral, showed strombine dehydrogenase and alanopine dehydrogenase activity but no LDH activity^25^. Another potential explanation for the poor results of the LDH assay could be the rich protein content of the complete culture medium (including 5% FBS) used to suspend the cells isolated from the coral fragments. Coral cell viability is reduced in the absence of serum^4^ but an alternative approach could use complete culture medium made with heat inactivated serum^26^. Because both these assays rely on normalizing the measurements to a reference to quantify cell viability, we did not pursue further optimization of the assays in this study.

One of the complications associated with in vitro coral research is limited cell attachment. Compared to adherent mammalian cell lines which attach to the substrate, coral cells have shown limited attachment to standard culture flasks and plates^4^. Here, we worked with mixed, unsorted cells; therefore, we increased cell attachment by coating the well plates with poly-d-lysine to reduce cell loss during aspiration. Without poly-d-lysine, cell counts dropped drastically and reached levels below critical mass. Poly-d-lysine had the side effect of trapping the TiO_2_ nanoparticles which resulted in the persistence of TiO_2_ agglomerates after exposure medium washing, which is visibly present in the brightfield images. The entrapment of TiO_2_ particles could have reduced their interactions with *P. damicornis* cells and affected the measured cytotoxicity. TiO_2_ nanoparticles have strong UV photoactive properties which have led to their increased use in paints, solar cells, and sunscreens and consequently their unintentional release in the environment^27^. The toxicity of TiO_2_ particle exposure in various freshwater (reviewed in^28^) and marine organisms (reviewed in^29^) has been tested, and the toxicity has been shown to increase with UV exposure due to photoactivation^28,29^. It is important to note that UV conditions are not always reported making it challenging to determine UV-enhanced cytotoxicity. The light requirements of reef-building corals intrinsically involve UV exposure, and we applied a 10 h light/14 h dark cycle (PAR 40 ± 2, i.e. ~ 8.71 W/m^2^ = 0.0871 mJ/cm^2^, see “Materials and methods” section for more details) throughout our experimentation. TiO_2_ particles, with particle sizes ranging from 20 to 200 nm haves been shown to damage symbiotic dinoflagellates and induce bleaching in *Acropora* spp. corals^30^ and *Montastraea faveolata*^31^. In both *Acropora* and *Montastraea*, slight bleaching occurred after exposure to 6.3 mg/L TiO_2_ for up to 48 h and 10 mg/L TiO_2_ for 17 days. Our results corroborate these findings with a reduction in cell viability starting at 10 mg/L TiO_2_ with LC50 at 6.21 mg/L under a UV intensity five times lower than that experienced in the environment. Concentrations of TiO_2_ measured in seawater collected in coastal areas were reported to range between 0.02 and 0.9 mg/L of TiO_2_ per day based on a study performed along three beaches in the south of France^32^ and 7–40 µg/mL of TiO_2_ along three beaches of Majorca Island^33^ These concentrations, which were correlated to sunscreen use by beachgoers, are orders of magnitude less than the LC50 found here; however, studies to date including ours represent only short term exposures.

The symbiotic mutualism between coral host and dinoflagellate endosymbionts fulfills up to 90% of the holobiont’s energetic needs. This energy trafficking suggests a transport and signaling system where a molecule such as insulin could come into play. Furthermore, as bleaching involves the breakdown of symbiosis, this transport and signaling system can be disrupted, presumably with similarities to the diabetic response in vertebrates. Insulin production is recognized as an evolutionary ancient function and its presence has been demonstrated in many organisms in addition to humans (unicellular eukaryotes, fungi, worms, fruit flies). There are numerous reports of preproinsulin-like pseudogenes in a variety of different organisms including insects, invertebrates, plants and microbial eukaryotes and prokaryotes^34^. Remarkably, human insulin has been shown to have physiological effects on other organisms, such as *Acanthamoeba castellanii*^35^, suggesting a conservation of structure and function across long evolutionary distances. A genome-wide computational scan of *P. damicornis* predicted protein sequences, using Hhblits^36^ to look for remote homologues of human protein sequences, predicts that *P. damicornis* cells (host coral) have insulin and insulin receptors (see Supplementary Material S.3)^37^. To enable future investigation of this hypothesis, we investigated insulin cytotoxicity here as an example for proteotoxicity. Insulin has been a model system for the study of proteotoxicity in protein evolution^38^, and different conformations and oligomerization states of insulin are relevant in the etiology and treatment of diabetes^39,40^. Thus, the natural first step in evaluating the effects of insulin on *P. damicornis* is its potential cytotoxicity. Our findings suggest that insulin reduces *P. damicornis* cell viability (~ 18% decrease) at concentrations between 10 and 100 µg/mL. Insulin cytotoxicity is known to depend on different solvent properties, such as increased temperature and high concentrations of salts^41,42^, leading to insulin aggregates and misfolding. The salinity or the ionic strength of seawater could have had similar effects despite the relatively low temperature (25 °C) and basic nature of the culture medium. Thus, it is possible that seawater might be modifying insulin conformation and cytotoxic behavior, and the present work lays the foundation for further research related to the effects of insulin on corals.

The insulin concentration in the serum used was unfortunately not available however, based on similar products, we estimate the insulin from the FBS used contributed less than 0.04% of the lowest concentration tested (0.5 µg/mL) for cytotoxicity in this study.

Regardless of the organism studied, the type of cell death mechanism is highly dependent on the nature and duration of the stress applied and the ability of cells to maintain homeostasis. The positive control, Triton X-100, is a common surfactant used to lyse cells. It is cytotoxic to a number of cell types: ciliated protozoan, fish and mammalian^43^. Using it as a positive control for membrane integrity assays involves the understanding that cell count will decrease with increasing Triton X-100 concentration. Different mechanisms of cell death can be involved when coral cells are exposed to substances such as insulin and TiO_2_ NPs tested here. Therefore, the Triton X-100 serves as a positive control verifying that the assay worked and additional research is needed to identify the mechanisms of cell death. For example, in mammalian cells, the absence of caspase activation, cytochrome c release, DNA fragmentation, membrane damage and changes in cell morphology are all cell parameters that can be used to discriminate cell necrosis from apoptosis^44^ and other types of cell death. This distinction is also particularly relevant in the study of dysbiosis. The breakdown of symbiosis between endosymbiotic dinoflagellate algae and coral host is still not well understood^45^ despite the urgent need to characterize coral bleaching at the cellular level. Stable cultures of the endosymbiont-holding gastrodermal cells, combined with cytotoxicity and cell death mechanism assays could help better define the mechanisms of coral-dinoflagellate dysbiosis.

## Conclusion

The global loss of coral cover driven by anthropogenic climate change has underscored the need for a better understanding of coral cell biology. Research progress in this area will be driven by the optimization of marine invertebrate cell culture methods and the potential transfer of in vitro methods developed for model organisms. Cell viability is a key measurement, and development of membrane integrity assays for coral cell culture represents an essential step in the advancement of in vitro coral research. Our results show that the Hoechst-SYTOX dye pair is well suited for membrane integrity assessments in *P. damicornis* cells. This dye pair was used to measure quantitatively the toxicity of Triton X-100, TiO_2_ and insulin in coral cells for the first time in vitro. These results open the door to in-depth, quantitative evaluation of toxicity of different reagents on corals at the cellular level.

In addition, further development of cellular assays will provide new tools to better understand cnidarian cellular physiology, transmembrane exchanges and cell death mechanisms.

## Methods

### Coral cell dissociation and culture

Fragments of *Pocillopora damicornis* (ORA Aquaculture purchased from Live Aquaria, FL) were maintained in 37.8L aquaria and supplied with oxygenated artificial seawater (ASW, constant bubbling, reverse osmosis deionized water [5 Stage Premium RO/DI water saver system from Bulk Reef Supply] + Fritz Reef Pro Mix salts, SG 1.025 ± 0.002, pH 7.5–8, 25 °C ± 1 °C, pump flow rate: 378.5 L/H, pump filter: activated carbon Aqueon, 10 h-light/14 h-dark cycle AI Prime 16HD Reef light: blue 28%, royal 28%, green 28%, deep red 28%, UV 28%, violet 28%, cool white 28%, moonlight 28%, PAR 40 ± 2). The spectrum of the AI Prime 16HD light ranges from 380–700 nm with the highest intensity peak wavelength being 450 nm at peak PAR of 100 Mol (https://www.aquaillumination.com/products/prime). The LED wavelengths are as follows: blue 460–490 nm, royal blue 450 nm, green 520–550 nm, deep red 660 nm, UV 400 nm, violet 415 nm (note: no wavelength available for the moonlight LEDs). General weekly maintenance included tank cleaning to remove macroalgae overgrowth, coral feeding (live Artemia: Carolina brine shrimp eggs) and replacing 2L of seawater.

The cell dissociation protocol follows that reported in Roger et al.^4^ Briefly, a coral nubbin (*P. damicornis*) of ~ 5 mm length was cut using sterile clippers and placed in artificial seawater (RO/DI H_2_O + Fritz Reef Pro Mix) with Reef Dip coral disinfectant (25 µL/mL of seawater) under constant bubbling for 10 min. The nubbin was then rinsed with sterile filtered (0.22 µm pore polyethersulfone membrane to remove any potential particulates), autoclaved ASW 3 times and incubated in calcium- and magnesium-free ASW (autoclaved and sterile filtered) for 1 h in a biosafety cabinet under ambient light. The nubbin was then washed using the solution in the vial to detach remaining cells and maximize cell count. The cell suspension was centrifuged at 1200 rpm (204 RCF) for 3 min at 25 °C and the supernatant replaced with complete culture medium (CM). The complete CM is composed of 15% Dulbecco’s Modified Eagle Medium (without phenol red, with 17.491 M d-glucose, 2.50 mM l-glutamine) + 5% Fetal Bovine Serum + 0.5% antibiotic–antimycotic + 0.5% gentamicin + 79% filtered sterile ASW.

### TiO_2_ and insulin exposures

Dissociated coral cells in complete coral CM were plated in poly-d-lysine coated 96 well plates at a density of ~ 400,000 cells per well. Poly-d-lysine (ThermoFisher cat. No. A3890401) coated 96 well plates were prepared following the manufacturer’s protocol. Initial viability was assessed using a 1 mL aliquot of the cell suspension stained with the Hoechst 33342/SYTOX Orange live/dead staining solution and counted on a disposable hemocytometer (INCYTO C-Chip, Neubauer Improved format) prior to plating. Following a 1 h rest period (to allow for potential membrane repair^46^ and attachment to polylysine coated culture substrate^47^), cells were incubated in coral CM alone (negative control) or coral CM containing serial dilutions (0.5, 1, 5, 10, 50 and 100 µg/mL) of Triton X-100 (positive control, Sigma-Aldrich cat. no. T8787), insulin (human recombinant (yeast), MilliporeSigma cat. no. 11376497001), or TiO_2_ nanoparticles (nanopowder, 21 nm primary particle size, ≥ 99.5% trace metals basis, Sigma-Aldrich cat. no. 718467, characterization data presented in Supplementary Materials S.4) for 24 h at 25 °C. Triplicate wells were exposed for each dose and the assay was performed three times. After exposure, the plates were centrifuged at 1200 rpm (204 RCF) for 3 min at 25 °C. The medium was replaced by the Hoechst 33342/SYTOX Orange live/dead staining solution and incubated in the dark for 30–60 min. After staining the plates were centrifuged at 1200 rpm (204 RCF) for 3 min at 25 °C. The staining solution was aspirated and fresh coral CM was added to each well before imaging. Photos of each well (4× magnification) were taken using a Cytation3 microplate reader mounted with 4 filter cubes (BioTek Agilent): DAPI (λ_ex_ 350–405 nm, λ_em_ 415–480 nm), GFP (λ_ex_ 445-490 nm, λ_em_ 501–550 nm), RFP (λ_ex_ 505–560 nm, λ_em_ 520–570 nm) and Texas Red (λ_ex_ 575–600 nm, λ_em_ 510–585 nm). Cells were counted in each photo and percent viability post-exposure was determined by [(Hoechst positive cells—SYTOX positive cells)*100/(Hoechst positive cells)]. Cell counting was performed using BioTekGen5 software automation in 1000 × 1000 µm squares in each photo (five photos were taken in each well).

### Live/dead assay

Two fluorescent dyes were selected: Hoechst 33342 (20 mM, ThermoFisher cat. no. 62249) and SYTOX Orange (250 µM solution in DMSO, ThermoFisher cat. no. S34861). To a 1 mL coral cell suspension, 10 µL of SYTOX Orange (0.2 µM final concentration) and 2 µL of Hoechst 33342 (40 µM final concentration) were added. Hoechst 33342 and SYTOX Orange are both DNA-binding dyes; however, Hoechst 33342 is membrane permeable whereas SYTOX Orange is not. In other words, Hoechst 33342 stains all cells while SYTOX Orange stains only cells with damaged membranes. Cells were exposed to the staining solution for 30–60 min and washed with fresh complete coral CM before imaging.

### MTS and LDH assays

Dissociated coral cells were plated in 96 well plates at a density of ~ 250,000 cells per well in complete coral CM. After a 1 h rest period, initial viability was assessed using a small aliquot of the cell suspension stained with the Hoechst 33342/SYTOX Orange live/dead staining solution and counted on a disposable hemocytometer prior to plating. Cells were incubated in coral CM alone (negative control) or coral CM containing serial dilutions (0.5, 1, 5, 10, 50 and 100 µg/mL) of Triton X-100 (positive control), insulin, or TiO2 NPs for 4 h at 25 °C. Triplicate wells were exposed for each dose and the assay was performed three times. After exposure, the plates were centrifuged at 1200 rpm (204 RCF) for 3 min at 25 °C. A 50 µL volume of supernatant from each well was transferred to a white 96-well plate and left to equilibrate to room temperature for 20 min. Then, 50 µL of CytoTox-ONE reagent (CytoTox-ONE Homogeneous Membrane Integrity Assay kit, Promega, G7891) was added to each well and incubated at room temperature for 10 min. After adding 25 µL of Stop Solution to each well, the fluorescence intensity was measured at λ_ex_ 560/λ_em_ 590 nm using a microplate reader (Cytation 3, BioTek).

The remaining exposure medium was removed from the wells and 120 µL of MTS reagent was added to each well. The MTS reagent consisted of 7.1 mL of coral CM and 1.4 mL of MTS (CellTiter 96 AQueous One Solution Cell Proliferation Assay kit, Promega, G3580). The plate was incubated at 25 °C for 1 h in the dark. Following incubation, the absorbance was measured at 490 nm using a microplate reader.

### Statistical analysis

The cell viability data was analyzed using a two-way ANOVA with blocking to consider variability between replicate assays (viability ~ concentration + assay + molecule), and Tukey post-hoc (95% family-wise confidence level). The LC50 of Triton X-100, TiO2 NPs and insulin were calculated using the probit regression model in the R package ecotox^48^ v. 1.4.4 available on CRAN. All analyses performed and results can be found in Supplementary Materials S.2.

### Microscopy: laser scanning microscopy

Single optical sections of coral cell suspension were acquired with a Zeiss LSM 880 or LSM 710 confocal microscope, build on Axio Observer Z1 inverted stand, equipped with motorized stage and a 40× Plan Apo oil immersion objective (NA 1.4). Fluorescence was excited with either one or a combination of lasers available on the system: 405 nm diode (15 mW), 440 nm diode (15 mW), multiline Ar ion (458/488/514 nm, 25 mW), 561 nm DPSS (15 mW), 594 nm and 633 nm He–Ne (3 mW each). Excitation power was adjusted between 0.8% and 2%, depending on the line. Fluorescence spectrum was registered with a 32-channel spectral hybrid detector at the gain of 500 V. Each channel corresponded to 7.9 nm. The pinhole was set to 1.25 Airy units at 550 nm emission. Transmitted light (DIC) was registered together with fluorescence. Images were collected in tile-based mode (5 × 5), with total area of 1220 × 1220 um, 0.13 um pixel size and 2.05 us dwell time. All signals were digitized with 16-bit precision.

### Microscopy: microplate reader

The cytotoxicity assay plates were imaged (4× magnification) using a Cytation3 microplate reader mounted with 4 filter cubes (BioTek Agilent): DAPI (λ_ex_ 350–405 nm, λ_em_ 415–480 nm), GFP (λ_ex_ 445-490 nm, λ_em_ 501–550 nm), RFP (λ_ex_ 505–560 nm, λ_em_ 520–570 nm) and Texas Red (λ_ex_ 575–600 nm, λ_em_ 510–585 nm). Image analysis and cell counts were performed in the BioTekGen5 software.

### UV/VIS-fluorescence spectrophotometer

Cells isolated from *Pocillopora damicornis* (mixed cells: coral host and symbiotic algae) suspended in sterile artificial seawater were analyzed using a Varian Cary Eclipse Fluorimeter with the following settings: scan from λ_ex_ 200–700/λ_em_ 350–900, excitation slit 20 nm, emission slit 5 nm, scan rate 9600.00 nm/min, data interval 2.00 nm, average time 0.0125 s, auto excitation filter, open emission filter, medium PMT voltage, no corrected spectra. The resulting data was used to produce the excitation–emission matrix presented in Fig. 3.

## Supplementary Information

Supplementary Information 1.

Supplementary Information 2.

Supplementary Information 3.

Supplementary Information 4.

Supplementary Information 5.

## Data Availability

All spectral scans performed on *Pocillopora damicornis* mentioned in the Results section and Supplementary Materials S.1 are available on the Open Science Framework repository https://osf.io/69jpx/ (https://doi.org/10.17605/OSF.IO/69JPX). All *Pocillopora damicornis* protein sequences and human protein sequences analyzed in the Discussion and Supplementary Materials S.3 are available in UniProtKB https://www.uniprot.org/. The remainder of the data generated in this study are presented in the text or in Supplementary Materials.
