# Supplementary material for: Evaluation of fluorescence-based viability stains in cells dissociated from scleractinian coral Pocillopora damicornis
Source: Sci Rep. 2022 Sep 12;12:15297. doi: 10.1038/s41598-022-19586-7 (PMC9468155; doi:10.1038/s41598-022-19586-7)
Supplement: Supplementary file 1 — Supplementary Information 1. [file 41598_2022_19586_MOESM1_ESM.pdf]

Supplemental Materials

S.1. Spectral scans performed on *Pocillopora damicornis* live cells with seven different lasers: 405 nm (**A**); 440 nm (**B**); 458 nm (**C**); 488 nm (**D**); 514 nm (**E**); 561 nm (**F**); 633 nm (**G**). (Zeiss LSM 710, VCU Microscopy Core)

S.2. Cytotoxicity data and statistical analysis.

The first sheet of the excel file presents the cytotoxicity data of TiO_2 and insulin on *Pocillopora damicornis* cells (Triton X-100 as positive control) for doses from 0.5 to 100 µg/ mL after 24 hours of exposure.

The second sheet (sheet "ANOVA") presents the statistical analysis performed on the data (from the first sheet) using R studio.

The third sheet ("LC50") presents LC50 calculation parameters and results.

S.3. Remote sequence homology detection of insulin. HHblits was used to align the human and pdam sequences. Shown in different colors are various residue motifs identified to be important for receptor binding by cryoelectron microscopy.

S.4. Transmitted Electron Microscopy image of Titanium dioxide (TiO_2) powder and absorbance curve.
