# Supplementary material for: Evaluation of fluorescence-based viability stains in cells dissociated from scleractinian coral Pocillopora damicornis
Source: Sci Rep. 2022 Sep 12;12:15297. doi: 10.1038/s41598-022-19586-7 (PMC9468155; doi:10.1038/s41598-022-19586-7)
Supplement: Supplementary file 2 — Supplementary Information 2. [file 41598_2022_19586_MOESM2_ESM.docx]

**S.1**. Spectral scans performed on *Pocillopora damicornis* live cells with seven different lasers: 405 nm (**A**); 440 nm (**B**); 458 nm (**C**); 488 nm (**D**); 514 nm (**E**); 561 nm (**F**); 633 nm (**G**). (Zeiss LSM 710, VCU Microscopy Core): <https://osf.io/69jpx/>

DOI 10.17605/OSF.IO/69JPX
