# Supplementary material for: Evaluation of fluorescence-based viability stains in cells dissociated from scleractinian coral Pocillopora damicornis
Source: Sci Rep. 2022 Sep 12;12:15297. doi: 10.1038/s41598-022-19586-7 (PMC9468155; doi:10.1038/s41598-022-19586-7)
Supplement: Supplementary file 4 — Supplementary Information 4. [file 41598_2022_19586_MOESM4_ESM.pdf]

HHblits sequence alignment

```
Q sp|P01308|INS_     2 ALWMRLLPLLALLALWGPDPAA---------AFVNQHLCGSHLVEALYLVCGERGFFYTPKTRREAEDLQ--VGQVELGG   70 (110)
Q Consensus          2 alwmrllpllallalwgpdpaa---------afvnqhlcgshlvealylvcgergffytpktrreaedlq--vgqvelgg   70 (110)
                       .+|.-++.|+.|+++-....+.         .=..+++||++|+++|..||+.+++.+.++... ..+..  ...+.-
T Consensus          5 ~~~~~~~~l~~l~~~~~~~~~~~~~~~~~~~~~~~~~~CG~~L~~~L~~vC~~~~~~~~~~~~~~~-~~~~~~~~~~l~~--   81 (116)
T pdam_00006633-     5 LLWTIVPFLAIVLSLEAVTGSKLVKAYEVGSRRIDAHICGDHIKEVYTKVCIDESVGKRKRRSP-LMEEKEALSFIHS--   81 (116)
Confidence               3566666666665443222111         113589999999999999999887654443311 00000  000100

Q sp|P01308|INS_    71 GPGAGSLQPLALEGSLQKRGIVEQCCTSICSLYQLENYC  109 (110)
Q Consensus         71 gpgagslqplalegslqkrgiveqcctsicslyqlenyc  109 (110)
                        ..+.+   +...-.-++||||+.||..-|++.+|+.||
T Consensus         82 -~~~~~---~~~~r~~~~rGIvdECC~~~Ct~~eL~~YC  116 (116)
T pdam_00006633-    82 -ESNRS---LRKARSVRTVNIVEECCIEGCTIGELKEYC  116 (116)
Confidence                01111   1111222558999999999999999998
```

```
No 1
>pdam_00006633-RA
Probab=98.81  E-value=3.2e-13
Score=85.14  Aligned_cols=101
Identities=24%  Similarity=0.440
Sum_probs=58.9  Template_Neff=7.200
```

HHblits results

```
No 1

>pdam_00013976-RA protein Name:"Similar to HTK7 Putative insulin-like peptide receptor (Hydra vulgaris)" AED:0.13 eAED:0.14
QI:0|0.30|0.29|1|0.88|0.96|27|0|1306

Probab=100.00  E-value=1.1e-187  Score=1601.88  Aligned_cols=1164  Identities=42%  Similarity=0.757  Sum_probs=988.5  Template_Neff=6.200

Q sp|P06213|INSR   28 HLYPGEVCPGMDIRNNLTRL-HELENCSVIEGHLQILLMFKTRPEDFRDLSFPKLIMITDYLLLFRVYGLESLKDLFPNL  106 (1382)

Q Consensus        28 hlypgevcpgmdirnnltrl-helencsvieghlqillmfktrpedfrdlsfpklimitdylllfrvygleslkdlfpnl  106 (1382)

                      |.-.|.|-  .||||+...| ..|+||+||+|+|+|++|.+....+|++++||++-.||+|||+++++++++.++||||

T Consensus         8 ~~~~~~~~~--~~~~~~~~~~~~~~~~~nCt~i~G~l~i~~~~~~~~~~~~~~~~~f~~i~eItgylli~~~~~~~~~~~~~~f~nL   85 (1306)

T pdam_00013976-    8 HTTAGKVV--LKISNEKCDGCEKLENCTTLEGSIQVQMVRKASDAVMKQLQFPKLTEITGHLLVSLMYGRRSLREIFPNL   85 (1306)

Confidence            44444442  6999999999 99999999999999999887888899999999999999999999999999999999

Q sp|P06213|INSR  107 TVIRGSRLFFNYALVIFEMVHLKELGLYNLMNITRGSVRIEKNNELCYLATIDWSRILDSVE-DNY-IVLNKDDNEECGD  184 (1382)

Q Consensus       107 tvirgsrlffnyalvifemvhlkelglynlmnitrgsvrieknnelcylatidwsrildsve-dny-ivlnkddneecgd  184 (1382)

                      +||||..||.+|||||++|.+|++|||.+|..|.||+|+|++|..|||+-||||++|+++.. ++| ++.|+. .++|.+

T Consensus        86 ~vIrG~~l~~~~~al~v~~~~~~~l~~l~l~sL~~I~~G~V~i~~N~~LCy~~TI~W~~I~~~~~~~~~~~~~~~~~~~~-~~~~c~~  164 (1306)

T pdam_00013976-   86 AVIRGRQVFLDYSLIIYQNDGLEEVNLPSLTTILRGGVRIEKNINLCYVETIRWKSIMRNTKVDEYTLVLNSN-NNDCYD  164 (1306)

Confidence            99999999999999999999999999999999999999999999999999999999999998863 344 455555 668999

Q sp|P06213|INSR  185 ICPGTAKGKTNCPATVINGQFVERCWTH-----SHCQKVCPTICKSHGCTAEGL------CCHSECLGNCSQPDDPTKCV  253 (1382)

Q Consensus       185 icpgtakgktncpatvingqfvercwth-----shcqkvcptickshgctaegl------cchseclgncsqpddptkcv  253 (1382)

                      .||+..    -|+.+..++.+.++||..     .|||+.||..|..+||.+.|.      |||.||+|.|+.|+++++|+

T Consensus       165 ~c~~~~~~----~~~~~~~~~~~~~~~~~~~~CW~~g~~~~~~~~CQ~~C~~~C~~~~~~~~~~~~~~~~~~~~~~~~~~cCh~~C~ggc~~p~~~~~~C~  240 (1306)

T pdam_00013976-  165 RCFQQK----CTPPAGHGSLTNQYCWAPGAGSNADCQALCDMKCGDSGCVNGGLMGKSTSCCDKQCLGGCTKTNSPHHCY  240 (1306)

Confidence            999762    345566677788999964     89999999999999987765      8999999999999999999

Q sp|P06213|INSR  254 ACRNFYL-DGRCVETCPPPYYHFQDWRCVNFSFCQDLHHKCKNSRRQGCHQYVIHNNKCIPECPSGYTMNSSNLLCTPCL  332 (1382)

Q Consensus       254 acrnfyl-dgrcvetcpppyyhfqdwrcvnfsfcqdlhhkcknsrrqgchqyvihnnkcipecpsgytmnssnllctpcl  332 (1382)

                      +|+||++ ||.||++|||++|++.+||||.  .|.+-              |..++++||++||.||++.. +.-|++|-

T Consensus       241 ~C~~~~~~~~~~g~Cv~~Cp~~~~~~~~~~~~~~~~~Ci~---cp~~~~~~~~~~~~~~~~~~~~~~~~~~~~~~~~~~~~~~~Cv~~CP~gy~~~~~-~~~C~~C~  303 (1306)

T pdam_00013976-  241 ACRNFRMPKGECVEKCGPGLYEIDEFKCID--NCPDG--------------YLKLGMKCAKVCPAGYKEGG-NKSCLKCT  303 (1306)

Confidence            9999986 7999999999999999999996  34322              3467899999999999864 68999998
```

```
Q sp|P06213|INSR  333 -GPCPKVCHLLEGEKTIDSVTSAQELRGCTVINGSLIINIRGGNNLAAELEANLGLIEEISGYLKIRRSYALVSLSFFRK  411 (1382)
Q Consensus       333 -gpcpkvchllegektidsvtsaqelrgctvingsliinirggnnlaaeleanlglieeisgylkirrsyalvslsffrk  411 (1382)
                       +.|||-                                      ...|||++||.||||.|||+|++|.++++|.|||+
T Consensus       304 ~~~c~~~--------------------------------------~~~ele~~~~~~~~~~g~l~i~~s~~~~~~~~~~~  345 (1306)
T pdam_00013976-  304 TEKCPRG--------------------------------------IGTQLEENLGQIEKVNGYIVIIESASLTSLNFFKN  345 (1306)
Confidence           678873                                      26779999999999999999999999999999999
Q sp|P06213|INSR  412 LRLIR--------------GETLEIGNYSFYALDNQNLRQLWDWSKHNLTITQGKLFFHYNPKLCLSEIHKMEEVSGTKG  477 (1382)
Q Consensus       412 lrlir--------------getleignysfyaldnqnlrqlwdwskhnltitqgklffhynpklclseihkmeevsgtkg  477 (1382)
                      ||.||              |.+||-|+|+++.+|||||++||+|.+ +|+|.+|++|||||||||...|..+-.-++.-.
T Consensus       346 l~~i~~~~~~~~~~~~~~~~~~~~~~~~~~~~~~~~~~~~~~~~-~~~~~~~~~~~~~~~~lc~~~i~~~~~~~~~~~~~  424 (1306)
T pdam_00013976-  346 LREIRPRLIYNFLSRPPAMETDLYNERYALAIRDNPKLEALWPFQQ-NLTIIEGGIMVHLNPYLCPSQITPLINDILKWN  424 (1306)
Confidence          99998              456778999999999999999999998 999999999999999999988887766544433
Q sp|P06213|INSR  478 RQ--ER-NDIALKTNGDQASCENELLKFSYIRTSFDK------ILLRWEP-YWPPDFRDLLGFMLFYKEAPYQNVTEFDG  547 (1382)
Q Consensus       478 rq--er-ndialktngdqascenellkfsyirtsfdk------illrwep-ywppdfrdllgfmlfykeapyqnvtefdg  547 (1382)
                      |.  ++ -|++-++|||..+|.-..|.+.--..+--+      ++++|++ ---.+.+.++|+.+.|+|+|+++||.++|
T Consensus       425 ~~~~~~~~~~~~~~~~~~~~c~~~~~~~~~~v~~~~~~~~~~~~~~~v~~~w~~~~~~~~~~~~~~~y~i~~~~~~~~~~~~y~~  504 (1306)
T pdam_00013976-  425 RNDSNRVLDISDTTNGNAVACNVRKINVTVEEITLPRGCNPVCVKVEWDDAIINDDYRNVLFYTLSYREAPNRQITEYTD  504 (1306)
Confidence          33  23 489999999999999998887655444334      8999976 33346899999999999999999999999
Q sp|P06213|INSR  548 QDACGSNS---WTVVDIDPPLRSNDPKS-----QNHPGWLMRGLKPWTQYAIFVKTLVTFSDERRTYGAKSDIIYVQTDA  619 (1382)
Q Consensus       548 qdacgsns---wtvvdidpplrsndpks-----qnhpgwlmrglkpwtqyaifvktlvtfsderrtygaksdiiyvqtda  619 (1382)
                      .|+|.++.   |++.|+..+...+....     ...-...+.+|+|+|+|.+.|++.-...+     |..|+.++|.|+.
T Consensus       505 ~~~~~~~~~~~w~~~~~~~~~~~~~~~~~~~~~~~~~~~~~~i~~L~p~T~Y~~~V~A~n~~G~-----g~~S~~~~v~T~e  579 (1306)
T pdam_00013976-  505 VDACSSDSGDIWTRIDHTVPPPEVNVSRGLITKRRKIERTIKKLKPYQLYAFQVEAVVLKND-----GAKSDLVFVMTKE  579 (1306)
Confidence          99999877   99999887665553322     2445678999999999999999998655444     7889999999999
Q sp|P06213|INSR  620 TNPSVPLDPISVSNSSSQIILKWKPPSDPNGNITHYLVFWERQAEDSELFELDYCLKGLKLPSRTWSPPFESEDSQKHNQ  699 (1382)
Q Consensus       620 tnpsvpldpisvsnsssqiilkwkppsdpngnithylvfwerqaedselfeldyclkglklpsrtwsppfesedsqkhnq  699 (1382)
                      ..|+.|.+....+.+++.|.+.|+||..|||+|+.|.|+|.....+.+....+|+.+-      .++++-......++++
T Consensus       580 ~~Ps~P~~v~~~~~sst~i~v~W~pP~~~NG~I~gY~v~y~~~~~~~~~~~~~~~~~~~------~~~~~~~~~t~~~~~~~~  653 (1306)
T pdam_00013976-  580 SKPSQPVGLEANYLNSSALLVTWEPPLFPNGNITKYIVSYEISTYSAWKADLDWCSRQ------VFSNRLGGNNNGEGST  653 (1306)
```

```
Confidence             9999999988888999999999999999999999999999999999988888888774      3455566667778889

Q sp|P06213|INSR  700  SEYEDSAGE----CCSCPKTDSQILKELEESSFRKTFEDYLHNVVFVPRKTSSGTGAEDPRPSRKRRSLGDVGNVTVAVP  775 (1382)

Q Consensus       700  seyedsage----ccscpktdsqilkeleessfrktfedylhnvvfvprktssgtgaedprpsrkrrslgdvgnvtvavp  775 (1382)

                       ...+|..+|    ||+|+|.+.++.+|.++++|+|||||||.||.||            |+|++|.+.++.+.|-|.|

T Consensus       654  ~~~pd~~~~~~~~c~~~~~~~~~~~~~~~~~~~~~~~~fe~~~~~~~~~~~~~~~~~------------~~~~~~~~~~~~~~~~~~  721 (1306)

T pdam_00013976-  654  DNTPDGNCETNLTCTCDDKEEEKMKPEKQSALFAKEFQDILYKTLFTKT------------EDDNEPPTNKTVLATTAPT  721 (1306)

Confidence             999999998    999999999999999999999999999999987            8889999999999999999

Q sp|P06213|INSR  776  TVAAFPNTSSTSVPT------------------------SPEEHRPFE------KV-VNKESLVISGLRHFTGYRIELQA  824 (1382)

Q Consensus       776  tvaafpntsstsvpt------------------------speehrpfe------kv-vnkeslvisglrhftgyrielqa  824 (1382)

                       ++..+||.+++..+.                        +-...-||.      +| -++|..+|++|||||-|+|+++|

T Consensus       722  ~~~~~~~~~~~~~~~~~~~~~~~~~~~~~~~~~~~~~~~~~~~~~~~~~~~~~~~~~~~~~~~~~~~~~~l~~ft~y~i~v~a  801 (1306)

T pdam_00013976-  722  TGLNQPNCSNNAQNPMCQPTTAPSGKKTTKLSSTSVKPTSMRPTVPATKPNASLTVDGNVNKIPLTNLRHFSDYTITVCA  801 (1306)

Confidence             998888877653211                        111111222      22 26789999999999999999999

Q sp|P06213|INSR  825  CNQDTPEERCSVAAYVSARTMPEAKADDIVGPVTHEIFENNV------VHLMWQEPKEPNGLIVLYEVSYRRYGDEELHL  898 (1382)

Q Consensus       825  cnqdtpeercsvaayvsartmpeakaddivgpvtheifennv------vhlmwqepkepnglivlyevsyrrygdeelhl  898 (1382)

                       |+...    |+|...+++++    .+|||.|+.+|+..++++      |+|-|++|..|||-=-=         -+.-.+

T Consensus       802  ~~~~~------------------~~~~~~~~~~~~~~~~~~~~~~~~~~~~~~~~~~~~~n~~~~~~~-------------~~~~~  864 (1306)

T pdam_00013976-  802  CTKVG----CATGSSCATTK----GMTNKNGSRQIIKIFLCIISATVSIIMGFPKGPKWSGAQSD---------SQPEFK  864 (1306)

Confidence             99876    66666666666    67999999999999995      99999999999994211         122346

Q sp|P06213|INSR  899  CVSRKHFALERGCRLRGLSPGNYSVRIRATSLAGNGSWTEPTYFYVTDYLD--VP-----SN--IAKIIIGPLIFVFLFS  969 (1382)

Q Consensus       899  cvsrkhfalergcrlrglspgnysvriratslagngswteptyfyvtdyld--vp-----sn--iakiiigplifvflfs  969 (1382)

                       |+|++...     ..-..-+|+|+.+++|++.+|+|+|+.+..|.+-..-.  .|     ..  ++.|++..+++++++.

T Consensus       865  c~s~~~~~~----------~~~~~~~~~~y~~~v~ais~~g~gs~~~~~~~~~~~~~~~s~~~~~~~~~~~~~~~~~~l~~Iig~~l~~~Vl~v  939 (1306)

T pdam_00013976-  865  CVSGKELK-----YQEKVEDGNYSAQVRAITSSGNGSWSNTVSFSYFIESQSTVPPIGEKDALSLGTIVGASLAASVVFV  939 (1306)

Confidence             87776432     1115678999999999999999999987665432111  11     11  1222222222222222

Q sp|P06213|INSR  970  -VVIGSIYLFLRKRQ---PDGP-LGPLYASSNPEYLSASDVFPCSVYVPDEWEVSREKITLLRELGQGSFGMVYEGNARD 1044 (1382)

Q Consensus       970  -vvigsiylflrkrq---pdgp-lgplyassnpeylsasdvfpcsvyvpdewevsrekitllrelgqgsfgmvyegnard 1044 (1382)

                        +++..+|++.|+|.   .+.. .+..+++.++++..+.+     ++.+++|++.++++++.+.||+|+||.||.|..++

T Consensus       940  lvv~llv~~i~r~r~kr~~~~~~~~~~~~~~~~~~~~~~~~~~~~~----~~~~~~~ei~~e~~~l~~~LG~G~FG~VYkg~l~~ 1014 (1306)
```

```
T pdam_00013976-  940 LACGFVVWYIARQRYKRYSDEQIPGVLYASVNPEYMTSTD-----VYIPDEWEFPREKIKLVRELGNGSFGMVYEGEAED 1014 (1306)

Confidence             22233444444332   2222 24456777777776555     46789999999999999999999999999999998

Q sp|P06213|INSR 1045 IIKGEAETRVAVKTVNESASLRERIEFLNEASVMKGFTCHHVVRLLGVVSKGQPTLVVMELMAHGDLKSYLRSLRPEAEN 1124 (1382)

Q Consensus      1045 iikgeaetrvavktvnesaslrerieflneasvmkgftchhvvrllgvvskgqptlvvmelmahgdlksylrslrpeaen 1124 (1382)

                      ...+....+||||+.++.++.+++.+|++|+.+|+.+..+|+|+++|+...+++.++|||+|..|+|.++|+..++.

T Consensus      1015 ~~~~~~~~~VAVK~l~~~~s~~~~~efl~Ei~iL~~L~HpNIV~LlG~~~~~~~~~LVmEym~~GsL~~~L~~~~~~--- 1091 (1306)

T pdam_00013976- 1015 ITPGDPMRRVAVKTVSENASIRDRVEFLQEASVMKQFCSNHVVRLLGVVSEGQPTLVIMELMELGDLKNFLRSRRPG--- 1091 (1306)

Confidence            88788888999999999888899999999999999999999999999999999999999999999999875543

Q sp|P06213|INSR 1125 NPGRPPPTLQEMIQMAAEIADGMAYLNAKKFVHRDLAARNCMVAHDFTVKIGDFGMTRDIYETDYYRKGGKGLLPVRWMA 1204 (1382)

Q Consensus      1125 npgrppptlqemiqmaaeiadgmaylnakkfvhrdlaarncmvahdftvkigdfgmtrdiyetdyyrkggkgllpvrwma 1204 (1382)

                      ....++.++++++++|.+||.|++|||.+.||||||||+||++.+++++||+|||+++...+.++++.++++..+.+|||

T Consensus      1092 ~~~l~~ls~~~~l~ia~qIA~GL~yLH~~gIVHrDIKpsNILLd~dg~~KLsDFGLAr~l~~~~~~~~~~~~~~t~~YmA 1171 (1306)

T pdam_00013976- 1092 EGTLPPPTLQELLQMAGEIADGMAYLAARKYVHRDLAARNCMVNADFTVKIGDFGMTRDIYETDYYRKGGKGLLPVRWMA 1171 (1306)

Confidence            23456778899999999999999999999999999999999999999999999999999999999999999999999999

Q sp|P06213|INSR 1205 PESLKDGVFTTSSDMWSFGVVLWEITSLAEQPYQGLSNEQVLKFVMDGGYLDQPDNCPERVTDLMRMCWQFNPKMRPTFL 1284 (1382)

Q Consensus      1205 peslkdgvfttssdmwsfgvvlweitslaeqpyqglsneqvlkfvmdggyldqpdncpervtdlmrmcwqfnpkmrptfl 1284 (1382)

                      ||.+..+.++.++||||||++|||+......||++.+++++++.+++++....|..+|+.+.+||+.||+.||++|||+.

T Consensus      1172 PE~l~~~~~t~ksDVySfGvvL~ELlTg~~~P~~~~s~~eil~~I~~g~~~~~p~~~p~~l~~LI~~C~~~dPs~RPS~~ 1251 (1306)

T pdam_00013976- 1172 PESLKDGVFTSYSDVWSFGVVMWEMATLAAQPYPGKSNEEVLKYVVDGGVMEKPEECPDRLYELMTLCWQFTTKARPTFV 1251 (1306)

Confidence            99999999999999999999999999999999999999999999999999999999999999999999999999999999

Q sp|P06213|INSR 1285 EIVNLLKDDLHPSFPEVSFFHSE 1307 (1382)

Q Consensus      1285 eivnllkddlhpsfpevsffhse 1307 (1382)

                      ++++.|.+++...|....|+++-

T Consensus      1252 eLl~~Le~~L~~~~~~~~~~~~~L 1274 (1306)

T pdam_00013976- 1252 FLISVLENDLSEDFRHLSFYHTL 1274 (1306)

Confidence            99999999999887777666654
```
