# Supplementary figures and images for: Evaluation of fluorescence-based viability stains in cells dissociated from scleractinian coral Pocillopora damicornis
Source: Sci Rep. 2022 Sep 12;12:15297. doi: 10.1038/s41598-022-19586-7 (PMC9468155; doi:10.1038/s41598-022-19586-7)

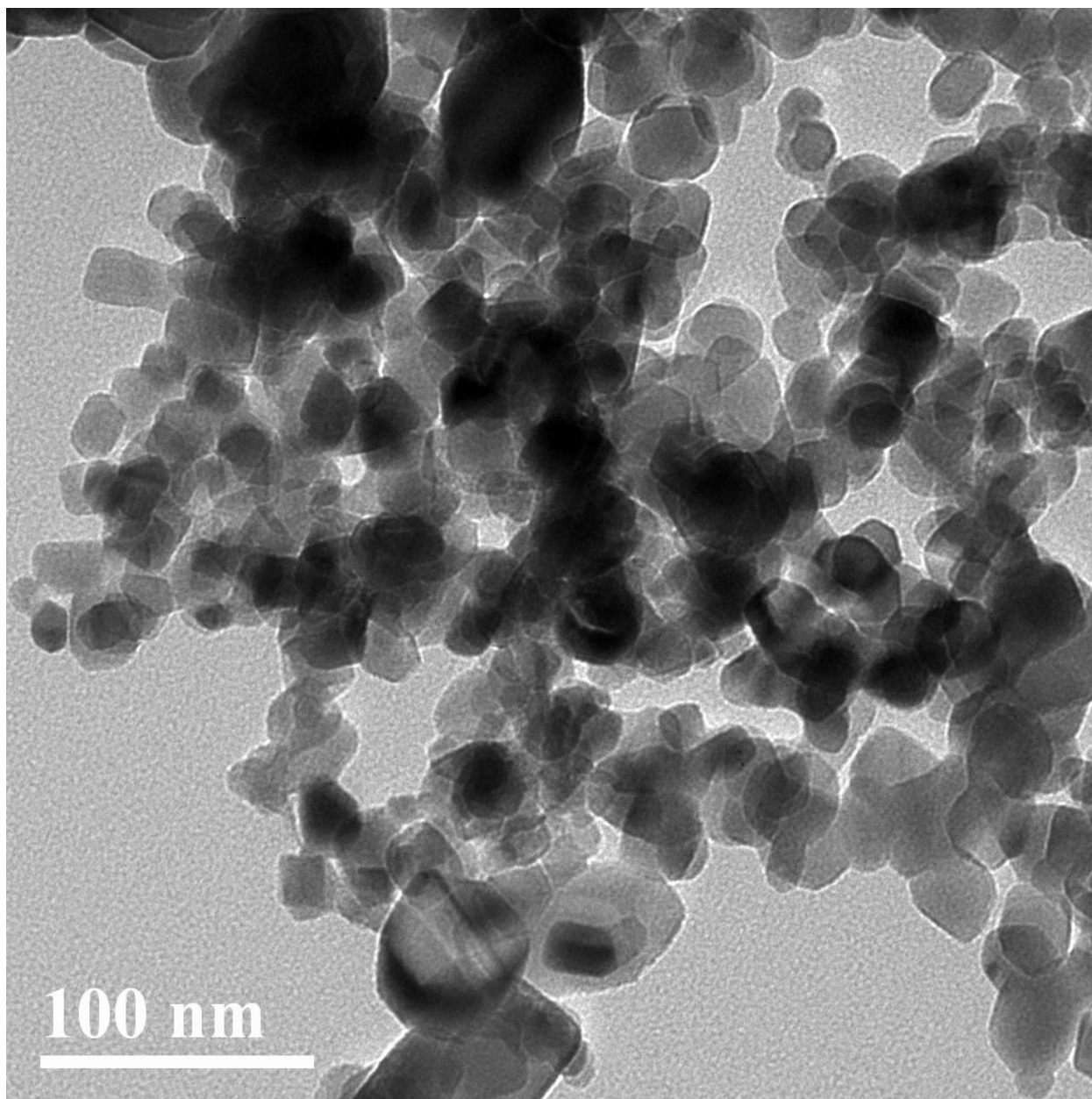

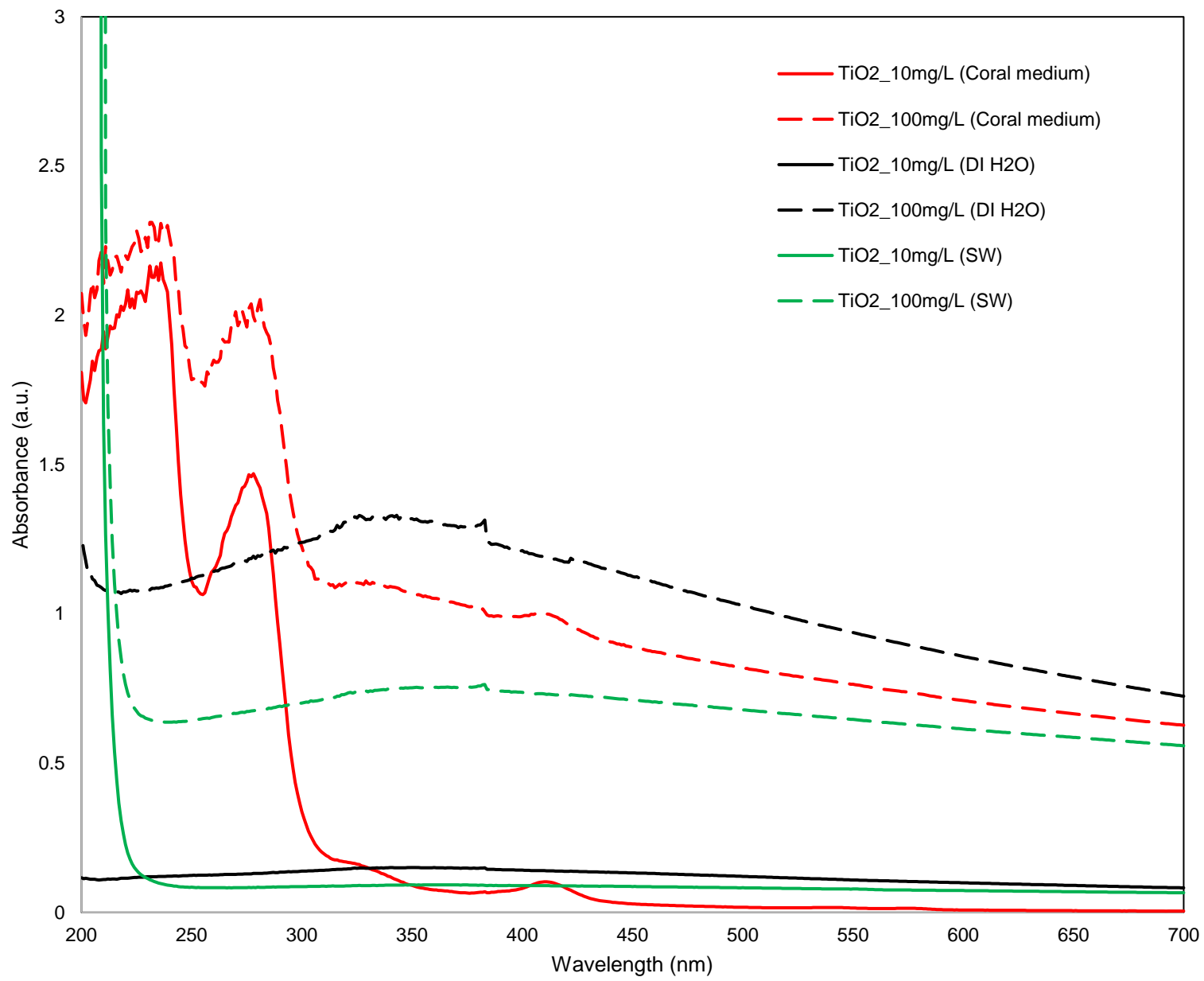

Supplement: Supplementary file 5 — Supplementary Information 5. [file 41598_2022_19586_MOESM5_ESM.pdf]
